# Respiratory failure among patients with COVID-19 in Jiangsu province, China: a multicentre retrospective cohort study

**DOI:** 10.1017/S0950268821000157

**Published:** 2021-01-20

**Authors:** Y. Wang, H. Luo, S. Liu, T. Hao, K. Mortimer, Y. Yang, D. Wang, S. Ju

**Affiliations:** 1 Department of Radiology, Zhongda Hospital, School of Medicine, Southeast University, Nanjing 210009, China; 2 Department of Clinical Sciences, Liverpool School of Tropical Medicine, Liverpool L3 5QA, UK; 3 Department of Critical Care Medicine, Jiangsu Provincial Key Laboratory of Critical Care Medicine, Zhongda Hospital, School of Medicine, Southeast University, Nanjing 210009, China

**Keywords:** 2019-nCoV, coronavirus, COVID-19, pneumonia, respiratory failure

## Abstract

This study was a retrospective multicentre cohort study of patients with coronavirus disease 2019 (COVID-19) diagnosed at 24 hospitals in Jiangsu province, China as of 15 March 2020. The primary outcome was the occurrence of acute respiratory failure during hospital stay. Of 625 patients, 56 (9%) had respiratory failure. Some selected demographic, epidemiologic, clinical and laboratory features as well as radiologic features at admission and treatment during hospitalisation were significantly different in patients with and without respiratory failure. The multivariate logistic analysis indicated that age (in years) (odds ratio [OR], 1.07; 95% confidence interval [CI]: 1.03–1.10; *P* = 0.0002), respiratory rate (breaths/minute) (OR, 1.23; 95% CI: 1.08–1.40; *P* = 0.0020), lymphocyte count (10^9^/l) (OR, 0.18; 95% CI: 0.05–0.69; *P* = 0.0157) and pulmonary opacity score (per 5%) (OR, 1.38; 95% CI: 1.19–1.61; *P* < 0.0001) at admission were associated with the occurrence of respiratory failure. Older age, increased respiratory rate, decreased lymphocyte count and greater pulmonary opacity score at admission were independent risk factors of respiratory failure in patients with COVID-19. Patients having these risk factors need to be intensively managed during hospitalisation.

## Introduction

The major clinical effects of coronavirus disease 2019 (COVID-19) infection are on the respiratory system although other systems can be affected [1, 2]. COVID-19 may result in acute respiratory failure requiring mechanical ventilation and even leading to death [3–6]. A study in Italy reported rates of respiratory failure as high as 29%–40% [7]. The 28-day mortality could occur in 26%–30% of patients with COVID-19 who had respiratory failure necessitating invasive mechanical ventilation (IMV) [8]. Since the COVID-19 pandemic is still evolving, the true mortality has not been defined, but the crude mortality ratio (the number of reported deaths divided by the number of reported cases) has been estimated to be 3%–4%, which appears to be higher than that for influenza [9]. However, the infection mortality rate (the number of reported deaths divided by the number of infections) is lower than the crude mortality ratio; and the mortality rate varies among different regions, demographic and socioeconomic characteristics, levels of healthcare access and quality, intervention methods and qualities of reported deaths and cases [9–11]. Identifying risk factors of respiratory failure in patients with COVID-19 could help clinicians recognise patients at high risk of respiratory failure and hence take active treatment for them to prevent further worse outcomes and reduce emergency intubation or cardiopulmonary resuscitation to protect medical staff from related infections.

For patients with COVID-19, risk factors of severe COVID-19, admission to intensive care unit (ICU) and death have been reported in many studies [12–15], but studies on risk factors of respiratory failure remain scarce. To the best of our knowledge, only two studies are available in the literature identifying the predictors of respiratory failure and both studies were conducted in Italy [7, 16]. However, demographic characteristics may vary across different countries, and the levels and risk factors of respiratory failure may also differ [17]. Therefore, information on the respiratory failure in other settings than Italy will be of great academic and clinical value.

Based on a large multicentre retrospective cohort with rich information on demographic, epidemiologic, clinical and laboratory features as well as CT imaging features, this study aims to assess the level and identify potential risk factors of respiratory failure in patients with COVID-19.

## Methods

### Study design and participants

The study design and participants have been described in our previous study [18]. Here we briefly describe it. This is a retrospective multicentre cohort study. Patient inclusion criterion was as of 15th March 2020, all patients diagnosed with COVID-19 at 24 hospitals designated to treat COVID-19 in Jiangsu province, China according to the diagnostic criteria of the ‘Diagnosis and treatment protocol for novel coronavirus pneumonia (trial version 7)’ released by National Health Commission and National Administration of Traditional Chinese Medicine of China [19]. The diagnosis of COVID-19 was based on epidemiological history, clinical and CT manifestations and laboratory confirmation (real-time reverse transcriptase-polymerase chain reaction assays [RT-PCR] to detect aetiological agent severe acute respiratory syndrome coronavirus-2, SARS-CoV-2, which caused COVID-19) [11, 19]. The exclusion criterion was patients with no available medical records. The criteria for discharge were: the patient's body temperature remained normal for more than 3 days, the symptoms were relieved (if there were symptoms), and the results of two consecutive RT-PCR assays were negative (throat swab samples, at least 1 day apart).

### Data collection and definition of variables

The primary outcome was the occurrence of acute respiratory failure during 14-day follow-up (days 1, 2, 3, 4, 5, 6, 7 and 14 from admission). The respiratory failure was defined as oxygen saturation (SpO_2_) <93% and/or partial pressure of oxygen in arterial blood (PaO_2_) <60 mmHg on room air and/or requirement of high-flow nasal cannula oxygen therapy (HFNC), non-invasive or invasive mechanical ventilation. Type 1 (hypoxaemic) respiratory failure refers to the hypoxaemia (PaO_2_ <60 mmHg (8 kPa)) with the normal (normocapnia) or low (hypocapnia) partial pressure of carbon dioxide (PaCO_2_) in arterial blood. Type 2 (hypercapnic) respiratory failure refers to the hypoxaemia with the hypercapnia (PaCO_2_ >45 mm Hg (6 kPa)).

Demographic features analysed in this study included sex and age (year). Epidemiologic features included exposure types (imported case or local case) and types of disease onset (single onset or clustered onset). Clinical features included initial symptoms (fever, cough, sputum, shortness of breath, anorexia and diarrhoea), medical history (hypertension, coronary heart disease, diabetes, stroke, smoking and drinking) and vital signs (temperature (°C), heart rate (HR, beats/minute), systolic blood pressure (SBP, mmHg), diastolic blood pressure (DBP, mmHg), respiratory rate (breaths/min) and SpO_2_ (%)). Laboratory features included blood test parameters (white blood cell count (WBC, 10^9^/l), neutrophil count (10^9^/l), lymphocyte count (10^9^/l), haemoglobin (g/l) and platelet count (10^9^/l)), organ function parameters (albumin (g/l) and creatinine (μmol/l)), inflammatory factors (C-reactive protein (mg/l)) and coagulation function parameters (activated partial thromboplastin time (APTT, s), fibrinogen (g/l) and D-dimer (mg/l)). Radiologic features included lesion distribution (outer third of lung involved, middle third of lung involved or inner third of lung involved), lesion density (below 20% consolidation, 20−80% consolidation, or above 80% consolidation), lesion border (well-defined border, moderately defined border or ill-defined border), quadrant score and pulmonary opacity score. Data of features listed above were collected at admission. Data of treatments (supportive treatments and medication) were obtained within the whole study period.

Imported cases were defined as those who had been to the pandemic centre of China (Wuhan city), or had contact with people or patients with COVID-19 who had been to Wuhan; and other cases were classified as ‘local cases’. A clustered onset is defined as the occurrence of two or more confirmed COVID-19 cases in the same cluster/group within 14 days, such as family, community, hospital, workplace or public place. A clustered onset may occur from interpersonal transmission via close contact with or joint contact with a confirmed COVID-19 case. Other cases not meeting the criteria for a clustered onset were classified as ‘single onset’.

Radiologic features were evaluated visually, by two radiologists with more than 5 years’ working experience. The radiologists were blinded to patients’ other characteristics and would reach agreements on different assessments of radiologic features. Chest CT axial sections were divided into four quadrants (left, right, anterior and posterior) by drawing horizontal and vertical lines through the centre of the chest. The quadrant score was defined as the number of quadrants with pulmonary opacities extending from the proximal end to the distal end of the chest, ranging from 0 to 4; and pulmonary opacity score was defined as the percentage of pulmonary opacity area in the area of bilateral lungs, rounded to the nearest 5%.

### Statistical analysis

Normally distributed continuous variables and skewed distributed continuous variables of patients were reported as mean (standard deviation, s.d.) and median (interquartile range, IQR) by group (patients with and without respiratory failure) and compared using Student's *t* test or Mann−Whitney *U* test, respectively. Categorical variables were summarised using frequency and percentage and compared by *χ*^2^ or Fisher's exact test.

Logistic regression models were used to identify risk factors of having respiratory failure. Univariate logistic regression models were fitted first to evaluate associations between each variable measured at admission and respiratory failure on the complete cases (without missing value). Variables that were statistically significant in the univariate analysis were then included in the multivariate logistic regression model. In the multivariate analysis, missing covariates were imputed with multiple imputation using a Markov chain Monte Carlo simulation method with 10 iterations. A sensitivity analysis was performed on complete cases. Odds ratio (OR) of having respiratory failure for each variable was calculated along with 95% confidence interval (CI). The two-tailed *P* < 0.05 was considered as statistically significant. The analyses were performed using SAS 9.4 (SAS Institute).

## Results

Of the 721 suspected cases with possible COVID-19 in Jiangsu province from 10th January to 15th March 2020, 90 cases were excluded since RT-PCR tests showed negative results and six cases were excluded due to no available medical records. Finally, 625 cases (52.6% male; median age 46 years old (IQR 32–57)) in 24 hospitals were included for analysis, mainly from the Second Hospital of Nanjing, Suzhou Infectious Disease Hospital and Huai'an No. 4 People's Hospital (113 (18.1%), 86 (13.8%) and 79 (12.6%), respectively). The remaining cases were disproportionately from the other hospitals. The hospital to which a patient was admitted was mainly determined by geographic location. Of the 625 patients, 56 (9%) had respiratory failure, mainly type I (hypoxaemic). At the study end point (15th March 2020), no patients died and all patients were discharged from hospitals.

At admission to hospital, compared with patients without respiratory failure, those with respiratory failure were significantly older (mean age 59.70 *vs.* 42.93 years, *P* < 0.0001); were more likely to be single onset (62.5% *vs.* 48.3%, *P* = 0.0498); were more likely to have symptoms including fever (82.1% *vs.* 64.3%, *P* = 0.0074), cough (71.4% *vs.* 53.4%, *P* = 0.0110), sputum (42.9% *vs* 25.0%, *P* = 0.0064) and shortness of breath (12.5% *vs.* 2.3%, *P* = 0.0010); were more likely to have prior histories of hypertension (32.1% *vs.* 13.9%, *P* = 0.0014), coronary heart disease (7.1% *vs.* 1.6%, *P* = 0.0224) and diabetes (17.9% *vs.* 5.3%, *P* = 0.0015); had higher mean temperature (37.25 *vs.* 37.03 °C, *P* = 0.0347), greater mean heart rate (90.71 *vs.* 86.82 beats/min, *P* = 0.0387), greater mean respiratory rate (21.13 *vs.* 18.88 breaths/min, *P* < 0.0001) and lower mean SpO_2_ (95.27% *vs.* 97.92%, *P* < 0.0001) (Table 1).

**Table 1.** Epidemiological and clinical characteristics of patients at admission

			Respiratory failure		
Category	Characteristics	All (*N* = 625)	Yes (*N* = 56)	No (*N* = 569)	*P* value
Demographic	Male, *n*(%)	329(52.6%)	36(64.3%)	293(51.5%)	0.0700
	Age (year), mean(s.d.)	44.44(17.21)	59.70(13.55)	42.93(16.80)	<0.0001
Exposure type, *n*(%)	Imported case	219(35.0%)	23(41.1%)	196(34.4%)	0.3784
	Local case	406(65.0%)	33(58.9%)	373(65.6%)	
Types of disease onset, *n*(%)	Single onset	310(49.6%)	35(62.5%)	275(48.3%)	0.0498
	Clustered onset	315(50.4%)	21(37.5%)	294(51.7%)	
Initial symptoms, *n*(%)	Fever	412(65.9%)	46(82.1%)	366(64.3%)	0.0074
	Cough	344(55.0%)	40(71.4%)	304(53.4%)	0.0110
	Sputum	166(26.6%)	24(42.9%)	142(25.0%)	0.0064
	Shortness of breath	20(3.2%)	7(12.5%)	13(2.3%)	0.0010
	Anorexia	13(2.1%)	1(1.8%)	12(2.1%)	1.0000
	Diarrhoea	53(8.5%)	6(10.9%)	47(8.3%)	0.4516
Medical history, *n*(%)	Hypertension	97(15.5%)	18(32.1%)	79(13.9%)	0.0014
	Coronary heart disease	13(2.1%)	4(7.1%)	9(1.6%)	0.0224
	Diabetes	40(6.4%)	10(17.9%)	30(5.3%)	0.0015
	Stroke	9(1.4%)	2(3.6%)	7(1.2%)	0.1900
	Smoke	25(4.0%)	0(0.0%)	25(4.4%)	0.1547
	Drinking	30(4.8%)	1(1.8%)	29(5.1%)	0.5066
Vital signs, mean(s.d.)	Temperature (°C)	37.05(0.73)	37.25(0.90)	37.03(0.71)	0.0347
	HR (beats/min)	87.17(13.46)	90.71(15.24)	86.82(13.24)	0.0387
	SBP (mmHg)	128.83(15.66)	131.16(18.89)	128.60(15.31)	0.2430
	DBP (mmHg)	81.47(10.51)	78.95(9.67)	81.72(10.57)	0.0596
	Respiratory rate (breaths/min)	19.08(2.56)	21.13(5.03)	18.88(2.07)	<0.0001
	SpO_2_ (%)	97.68(1.99)	95.27(4.95)	97.92(1.16)	<0.0001

s.d., standard deviation; HR, heart rate; SBP, systolic blood pressure; DBP, diastolic blood; SpO_2_, peripheral capillary oxygen saturation.

Patients with respiratory failure had significantly lower median lymphocyte count (0.7*10^9^/l *vs.* 1.3*10^9^/l, *P* < 0.0001), median platelet count (155.5*10^9^/l *vs.* 186.5*10^9^/l, *P* = 0.0008) and median albumin levels (40.0 *vs.* 41.9 g/l, *P* = 0.0005). In addition, patients with respiratory failure had significantly higher median levels of C-reactive protein (40.6 *vs.* 10.0 mg/l, *P* < 0.0001), median fibrinogen levels (4.3 *vs.* 3.4 g/l, *P* < 0.0001) and median D-dimer levels (0.3 *vs.* 0.2 mg/l, *P* = 0.0004) (Table 2).

**Table 2.** Laboratory parameters at hospital admission

		*N*, median(IQR)			
			Respiratory failure		
Category	Parameters	All	Yes	No	*P* value
Blood test	WBC count (10^9^/l)	5134.9(3.9–6.2)	45,4.5(3.4–5.8)	4684.9(3.9–6.3)	0.1742
	Neutrophil (10^9^/l)	5073.0(2.2–4.0)	45,3.1(2.1–4.6)	4623.0(2.2–4.0)	0.3912
	Lymphocyte (10^9^/l)	5051.3(0.9–1.7)	45,0.7(0.5–0.9)	4601.3(1.0–1.8)	<0.0001
	Haemoglobin (g/l)	510,137.0(124.0–150.0)	43,129.0(121.0–142.0)	467,137.0(124.0–150.0)	0.0772
	Platelet (10^9^/l)	494,183.5(151.0–219.0)	40,155.5(121.5–188.5)	454,186.5(153.0–220.0)	0.0008
Organ function	Albumin (g/l)	480,41.4(38.0–45.1)	41,40.0(34.0–41.4)	439,41.9(38.0–45.6)	0.0005
	Creatinine (μmol/l)	476,63.9(51.0–79.0)	42,62.4(49.1–85.0)	434,64.0(51.0–78.8)	0.9785
Inflammatory factors	C-reactive protein (mg/l)	474,10.0(2.7–22.6)	39,40.6(7.6–106.6)	435,10.0(2.6–19.7)	<0.0001
Coagulation function	APTT (s)	513,32.2(28.0–37.2)	47,32.0(28.0–36.6)	466,32.2(28.0–37.3)	0.7284
	Fibrinogen (g/l)	4963.5(2.7–4.2)	46,4.3(3.0–6.1)	4503.4(2.7–4.2)	<0.0001
	D-dimer (mg/l)	4750.2(0.1–0.4)	44,0.3(0.2–1.1)	4310.2(0.1–0.4)	0.0004

IQR, inter-quartile range; WBC, white blood cell; APTT, activated partial thromboplastin time.

For visually evaluated CT features at hospital admission, patients with respiratory failure had significantly greater median of CT quadrant score (4.00 *vs.* 2.00, *P* < 0.0001) and median of pulmonary opacity score (52.50% *vs.* 20.00%, *P* < 0.0001) (Table 3).

**Table 3.** Visually evaluated CT features at hospital admission

			Respiratory failure, *n*(%) or median(IQR)		
Category	Measurements	All	Yes	No	*P* value
Lesion distribution^a^	Outer third of lung involved	400(82.6%)	39(84.8%)	361(82.4%)	0.6873
	Middle third of lung involved	278(57.4%)	27(58.7%)	251(57.3%)	0.8561
	Inner third of lung involved	123(25.4%)	17(37.0%)	106(24.2%)	0.0587
Lesion density^a^	Below 20% consolidation	205(42.4%)	21(45.7%)	184(42.0%)	0.6343
	20%−80% consolidation	89(18.4%)	10(21.7%)	79(18.0%)	0.5375
	Above 80% consolidation	116(24.0%)	9(19.6%)	107(24.4%)	0.4622
Lesion border^a^	Well-defined border	90(18.6%)	9(19.6%)	81(18.5%)	0.8589
	Moderately defined border	82(16.9%)	8(17.4%)	74(16.9%)	0.9320
	Ill-defined border	235(48.6%)	23(50.0%)	212(48.4%)	0.8365
Other findings^b^	Quadrant score (0–4)	2.00(1.00–4.00)	4.00(4.00–4.00)	2.00(1.00–4.00)	<0.0001
	Pulmonary opacity (%)	20.00(5.00–40.00)	52.50(35.00–75.00)	20.00(5.00–35.00)	<0.0001

CT, computer tomography; IQR, inter-quartile range.

a The total number of cases is 484 (43 respiratory failures and 441 no respiratory failures).

b The total number of cases is 496 (47 respiratory failures and 449 no respiratory failures).

Oxygen was delivered to patients with respiratory failure via nasal cannula (80.4%), simple face masks (23.2%), HFNC (39.3%), non-invasive mechanical ventilation (NIV) (53.6%), IMV (8.9%) and in prone position (30.4%) (Table 4). Patients with respiratory failure were more likely to receive supportive treatments and medications (all *P* < 0.05), except for the interferon.

**Table 4.** Clinical management during hospitalisation

			Respiratory failure		
Category	Clinical management	All (*N* = 625)	Yes (*N* = 56)	No (*N* = 569)	*P* value
Supportive treatments, *n*(%)	Inotropic and vasoconstrictive agents	5(0.8%)	5(8.9%)	0(0.0%)	<0.0001
	Nasal cannula	221(35.4%)	45(80.4%)	176(30.9%)	<0.0001
	Mask	14(2.2%)	13(23.2%)	1(0.2%)	<0.0001
	High-flow nasal cannula oxygen therapy	25(4.0%)	22(39.3%)	3(0.5%)	<0.0001
	Non-invasive ventilation	34(5.4%)	30(53.6%)	4(0.7%)	<0.0001
	Invasive mechanical ventilation	5(0.8%)	5(8.9%)	0(0.0%)	<0.0001
	Prone position	18(2.9%)	17(30.4%)	1(0.2%)	<0.0001
Medical drugs, *n*(%)	Traditional Chinese medicine	98(15.7%)	29(51.8%)	69(12.1%)	<0.0001
	Immunoglobulin	156(25.0%)	41(73.2%)	115(20.2%)	<0.0001
	Interferon	503(80.5%)	40(71.4%)	463(81.4%)	0.0787
	Antioxidants	152(24.3%)	30(53.6%)	122(21.4%)	<0.0001
	Glucocorticoid	142(22.7%)	48(85.7%)	94(16.5%)	<0.0001
	Thymosin	144(23.0%)	35(62.5%)	109(19.2%)	<0.0001
	Neurotrophic drugs	102(16.3%)	19(33.9%)	83(14.6%)	0.0009
	Any antibiotics	336(53.8%)	53(94.6%)	283(49.7%)	<0.0001
	Any antivirals	580(92.8%)	56(100%)	524(92.1%)	0.0258

Twenty variables at admission were found to be related to the occurrence of respiratory failure in univariate logistic regression analysis (Table 5). When they were included in the multivariate logistic regression model simultaneously, four variables were independently related to the occurrence of respiratory failure: age (in years) (OR, 1.07; 95% CI: 1.03–1.10; *P* = 0.0002), respiratory rate (breaths/min) (OR, 1.23; 95% CI: 1.08–1.40; *P* = 0.0020), lymphocyte count (10^9^/l) (OR, 0.18; 95% CI: 0.05–0.69; *P* = 0.0157) and pulmonary opacity score (per 5%) (OR, 1.38; 95% CI: 1.19–1.61; *P* < 0.0001).

**Table 5.** Factors associated with respiratory failure in patients with COVID-19: Results from logistic regression analysis

	Univariate analysis^a^		Multivariate analysis^b^	
Variables	OR (95%CI)	*P* value	OR (95%CI)	*P* value
Age (year)	1.07(1.05,1.09)	<0.0001	1.07(1.03,1.10)	0.0002
Clustered onset	0.56(0.32,0.99)	0.0453	1.11(0.45,2.71)	0.8202
Fever	2.55(1.26,5.16)	0.0092	1.69(0.60,4.71)	0.3176
Cough	2.18(1.19,3.98)	0.0113	1.20(0.46,3.16)	0.7081
Sputum	2.26(1.29,3.96)	0.0046	1.45(0.55,3.86)	0.4520
Shortness of breath	6.11(2.33,16.02)	0.0002	0.82(0.15,4.59)	0.8242
Hypertension	2.94(1.60,5.40)	0.0005	0.80(0.30,2.13)	0.6589
Diabetes	3.91(1.80,8.49)	0.0006	2.13(0.59,7.68)	0.2469
Coronary heart disease	4.79(1.43,16.08)	0.0113	4.60(0.78,27.20)	0.0925
Temperature (°C)	1.44(1.02,2.03)	0.0362	0.67(0.39,1.17)	0.1607
HR (beats/min)	1.02(1.00,1.04)	0.0394	1.00(0.96,1.03)	0.7733
Respiratory rate (breaths/min)	1.25(1.15,1.37)	<0.0001	1.23(1.08,1.40)	0.0020
Lymphocyte (10^9^/l)	0.02(0.01,0.07)	<0.0001	0.18(0.05,0.69)	0.0157
Platelet (10^9^/l)	0.99(0.98,1.00)	0.0026	1.00(0.99,1.01)	0.9590
Albumin (g/l)	0.92(0.87,0.96)	0.0007	1.02(0.93,1.11)	0.7318
C-reactive protein (mg/l)	1.02(1.01,1.03)	<0.0001	1.00(0.99,1.01)	0.6707
Fibrinogen (g/l)	1.88(1.50,2.37)	<0.0001	0.95(0.64,1.41)	0.8005
D-dimer (mg/l)	1.33(1.10,1.60)	0.0035	1.15(0.78,1.71)	0.4824
Quadrant score (0–4)	2.37(1.71,3.28)	<0.0001	0.80(0.46,1.40)	0.4331
Pulmonary opacity (per 5%)	1.40(1.29,1.52)	<0.0001	1.38(1.19,1.61)	<0.0001

OR, odds ratio; CI, confidence interval; HR, heart rate.

a Univariate analysis is based on the complete cases without missing value.

b Multivariate analysis is based on imputed values for missing data in lymphocyte, platelet, albumin, C-reactive protein, fibrinogen, D-dimer, quadrant score and pulmonary opacity using multiple imputation method.

The sensitivity logistic regression model with only above four significant variables was estimated on complete cases (without missing data), and these variables remained statistically significant.

## Discussion

This study assessed the level and risk factors of respiratory failure among patients with COVID-19. During the 14-day follow-up, 9% (56 out of 625) of patients with COVID-19 suffered from respiratory failure (mainly type I, hypoxaemic) in Jiangsu province, China. At the end of the study (15th March 2020), no patients died and all patients were discharged from hospitals. Among many factors explored in this study, four of them (older age, increased respiratory rate, decreased lymphocyte count and greater pulmonary opacity score) were identified as independent risk factors of respiratory failure after controlling for other confounders. Therefore, patients with such factors need to be carefully and thoroughly managed by physicians.

Jiangsu province is non-neighbouring with and geographically distant around 600 km from Hubei province, where Wuhan city (the epicentre of COVID-19 pandemic in China) is located in. The rate of respiratory failure in Jiangsu province is similar to the figure in the national report from China [20] but this rate is much lower than reported earlier in Wuhan city (26%–32%) [5, 21]. This may be due to the early responses and measures adopted by the Jiangsu provincial health authorities to deal with the disease during the pandemic, including more adequate medical resources, deeper understanding and better management of respiratory failure for patients with COVID-19 [11]. The rate of respiratory failure in this study is also lower than reported in other countries, including Italy (29%–40%) [7] and the United States (~14%) [17], which may result from variable population demographics [17], and the early identification and early treatment of patients at high risk of respiratory failure in Jiangsu province [11].

Our study found that age was associated with respiratory failure. This is consistent with previous studies reporting that middle-aged and elderly patients with COVID-19 were susceptible to respiratory failure [4, 7], and is also similar to the results for patients with severe acute respiratory syndrome (SARS) [22–24]. A previous study showed that the older population have a higher incidence of comorbidities and hence possible poorer immune response to COVID-19 and poorer clinical outcomes [25]. This study also found that patients with respiratory failure tended to have more comorbidities including hypertension, coronary heart disease and diabetes.

Increased respiratory rate as a risk factor of respiratory failure identified in this study is simple and convenient to apply for clinicians in their practice. This result is consistent with previous studies [7, 26].

In our study, reduced lymphocyte count at admission was strongly associated with respiratory failure. Previous studies also show that the lymphocyte count of COVID-19 patients admitted to the ICU continued to decline [27, 28], and the reduced lymphocyte count was a risk factor for respiratory failure [7]. Reduced lymphocytes may be part of the pathogenesis of COVID-19 [29, 30]. Therefore, more attention needs to be paid to patients whose lymphocyte count decline more severely. Laboratory abnormalities including lymphopenia have been previously reported in severe cases of other respiratory viral diseases, including SARS, Middle East respiratory syndrome (MERS) and influenza [22, 31–34].

All radiologic images collected in our study were CT which have the advantage of high-resolution transversal imaging and accurate display of the extent and range of lung lesions. The potential measurement bias and misclassification bias resulted from visual assessment of CT images were controlled by double assessments of CT images by two independent radiologists with more than 5 years’ experience in pulmonary imaging, and by discussion and reaching agreements on different assessment results. This study showed that the pulmonary opacity score, one of the radiologic features, was strongly and independently associated with respiratory failure in patients with COVID-19, indicating that the more severe abnormality of lung function is an important factor to identify patients at high risks of respiratory failure. Former studies demonstrate that CT lung lesions can predict death and ICU admission in patients with COVID-19 [13, 35]. On the other hand, this study shows that between patients with and without respiratory failure, there were no significant differences in the distribution of lung lesions (both were more likely to involve the outer third of lungs), lesion density (both were more likely to have less than 20% of consolidation of lungs) and lesion boundary definition (both were more likely to have the ill-defined border of lungs).

In Jiangsu province with adequate medical material and human resources, all COVID-19 patients with respiratory failure received ICU monitoring. In comparison, due to limited resources at disease outbreak in Spain, some comorbid patients with respiratory failure requiring mechanical ventilation and ICU treatment did not receive these treatments which were reserved for non-comorbid patients, hence a large number of patients with respiratory failure finally died [36]. For similar settings with limited resources, the experience in Jiangsu province may be beneficial to COVID-19 patients with respiratory failure: in Jiangsu, a large proportion of patients with respiratory failure conducted prone position (30%) and received HFNC (40%) or NIV (~50%), which help reduce the further use of IMV (~10%) and mortality (no death occurred and all patients were discharged at the end of the study). This confirms the findings from several previous small studies [37, 38].

This study addressed several limitations of previous studies by (1) adding radiologic features and several epidemiologic, clinical and laboratory features into analysis to reduce residual confounding; (2) using multiple imputation method to provide unbiased estimates of levels and risk factors and (3) conducting a sensitivity analysis to confirm that results from multivariate logistic regression analysis, from which our main conclusion was drawn, were insensitivity to missing data and hence were robust and credible. This study included 625 patients from 24 hospitals, i.e. nearly all patients in Jiangsu province, China (a province with a population of 80 million; only six cases were excluded due to missing medical records), to make the findings from this study be subject to less selection bias and be more generalisable to populations in similar settings.

Our study has several limitations. Firstly, this is a retrospective observational study and its results may be subject to measurement bias and information bias, and some unobserved confounders (e.g. obesity, gene cluster) [16, 39]. Secondly, some data on laboratory test were missing and hence fewer laboratory parameters were included in the analysis. Thirdly, we were unable to analyse the impact of medical management on respiratory failure including supportive treatments and medication, because of the chronological order and treatment information collected before, when, or after respiratory failure occurred.

## Conclusions

In conclusion, this large cohort study based on a representative sample of 625 patients with COVID-19 shows that the rate of respiratory failure in Jiangsu province, China (9%), was similar to the national level in China, but much lower than in Wuhan city (the epicentre of COVID-19 pandemic in China) and some other countries. The study has also identified four independent risk factors of respiratory failure in patients with COVID-19 including older age, increased respiratory rate, decreased lymphocyte count and greater pulmonary opacity score at admission. For successful control of mortality related to COVID-19, patients with COVID-19 having these risk factors need to be intensively managed during hospitalisation.

## Data Availability

The datasets used and/or analysed during the current study are available from the corresponding author on reasonable request.
